# Biochip-Based Identification of Mycobacterial Species in Russia

**DOI:** 10.3390/ijms252313200

**Published:** 2024-12-08

**Authors:** Danila Zimenkov, Vyacheslav Zhuravlev, Anastasia Ushtanit, Marina Filippova, Uliana Semenova, Natalia Solovieva, Maria Sviridenko, Anastasia Khakhalina, Svetlana Safonova, Marina Makarova, Elizaveta Gordeeva, Elena Guselnikova, Yakov Schwartz, Natalia Stavitskaya, Peter Yablonsky

**Affiliations:** 1 Center for Precision Genome Editing and Genetic Technologies for Biomedicine, Engelhardt Institute of Molecular Biology, Russian Academy of Sciences, 119991 Moscow, Russia; 2 Saint-Petersburg State Research Institute of Phthisiopulmonology of the Ministry of Healthcare of the Russian Federation, 191036 Saint-Petersburg, Russia; 3 The Moscow Research and Clinical Center for Tuberculosis Control of the Moscow Government Health Department, 107014 Moscow, Russia; 4 Federal State Budgetary Institution “Novosibirsk TB Research Institute” of the Ministry of Health of Russian Federation, 630040 Novosibirsk, Russia

**Keywords:** mycobacteria, NTM, nontuberculous mycobacteria, biochip, oligonucleotide array, *M. petersburgensis*, *M. moscowiensis*, *M. sibiricum*

## Abstract

Infections caused by nontuberculous mycobacteria (NTM) are rising globally throughout the world. The number of species isolated from clinical samples is steadily growing, which demands the implementation of a robust diagnostic method with wide specificity. This study was carried out in in 2022–2024 in three clinical antituberculosis centers in the biggest cities of Russia: Moscow, Saint Petersburg, and Novosibirsk. We developed the DNA hybridization assay ‘Myco-biochip’ that allows the identification of 79 mycobacterial species and analyzed 3119 samples from 2221 patients. Sixty-eight mycobacterial species were identified in clinics, including the three novel species phylogenetically related to *M. duvalii*, *M. lentiflavum*, and *M. talmoniae*. The identification of a close relative of *M. talmoniae* adds to the existence of separate clade between *M. terrae*, *M. triviale* complexes and other slow-growing *Mycobacteria*, which supports the thesis against the splitting of *Mycobacteria* into five separate genera. Adding to the list of potentially pathogenic species, we identified *M. adipatum* and *M. terramassiliense*, which were previously described as natural habitats. The diversity of acid-fast bacilli identified in TB-suspected persons was not limited to the *Mycobacteria* genus and also includes species from genera *Nocardia*, *Gordonia*, *Corynebacterium*, *Tsukamurella*, and *Rhodococcus* of the order *Mycobacteriales*. The revealed bacterial diversity in patients with suspected NTM-diseases requires the implementation of relevant species identification assays as the first step in the laboratory diagnostic pipeline.

## 1. Introduction

The incidence of infections caused by nontuberculous mycobacteria (NTM) is steadily growing throughout the world [1]. Persisting in nature and urban environments, mostly in water support systems [2], mycobacteria could infect both immunocompetent and immunocompromised people [3]. Risk factors are older age and gender [4,5], inherited or acquired immunodeficiencies [6,7,8], surgery intervention [9,10], and others [3,10,11,12,13]. NTM cause pulmonary, cutaneous, and disseminate forms of infection, with the former being highly similar to tuberculosis, requiring the differentiation of *M. tuberculosis* from other *Mycobacteria* at the diagnosis stage [14,15].

*Mycobacteria* is a heterogeneous genus with more than 200 species [16] that includes slowly and rapidly growing bacteria, further divided into clusters and complexes [17]. During the last decade, many new mycobacterial species have been discovered, and most of them have been identified in clinical samples from patients [18]. NTM infections in humans are mostly caused by species that belong to *M. avium* and *M. chelonae-abscessus* complexes [19]. The growing number of pathogenic species [20,21,22] and variable intrinsic resistance of different species and subspecies requires the development of identification methods with wide specificity [23].

Conventional microbiological and biochemical identification approaches are being substituted with MALDI-TOF [24] and molecular methods [17]. The most widely used identification method in mycobacteriology used worldwide is the complex of two line probe assays GenoType Mycobacteria CM, and AS (Hain Lifesciense) [25]. Many alternative molecular assays based on various diagnostic platforms such as multiplex PCR [26], real-time PCR [27,28], sequencing [29], and various hybridization assays [30,31,32] were also developed. Low-density DNA hybridization biochip technology allows the multiplex identification of a particular sequence in genome with single nucleotide specificity at a reasonable cost [33].

One of the important questions is the reliability of genome loci for species identification [34]. The widely used fragments of the 16S rRNA gene appear to be non-species specific [35], and the whole rRNA operon is a more accurate marker for classification within a genus [36]. Therefore, other more diverse genes are preferred for species identification: *rpoB* [31,32], ITS fragment [37,38], *hsp65* [39,40], and *secA1* [29]. Previously, several groups [41,42,43], including our laboratory [44], have demonstrated the suitability of the approach for the identification of mycobacterial species by the analysis of the *gyrB* gene.

We previously developed the biochip assay for the identification of mycobacterial species and tested it on a number of clinical isolates from Moscow and Saint Petersburg [44], and recently on a set of isolates from Bulgaria [45]. While the developed method allowed the identification of 35 mycobacterial species in 543 clinical isolates, the analysis of larger sets of isolates showed its limitations for the identification of rare species. Two main obstacles, the similar hybridization profiles for distinct species and the incorrect annotation of sequences could lead to misidentification, and thus, to the necessity to complement the analysis with the target sequencing [45]. On the other hand, the biochip approach had several advantages, particularly the use of the *gyrB* fragment for species delineation, which was specificity confirmed using the newest sequencing data. It was supposed that the assay probes set is likely to be updated and expanded based on a burst of genomic data occurred during the last 10 years from the development of the first variant. The number of oligonucleotide probes, which corresponds to the clinically significant mycobacterial species, had been increased from 60 to 144, expanding the sensitivity and specificity of the approach. The novel assay was tested in the three largest cities in Russia and 68 species were found, revealing the hidden diversity of *Mycobacteria* identified in clinical samples from TB-suspected patients.

## 2. Results

### 2.1. The Development of Biochip Test for Species Identification

**Figure 1:**
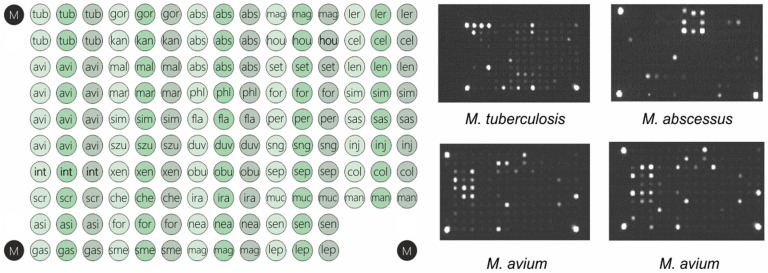
Myco-biochip layout scheme and examples of hybridization patterns. The list of oligonucleotide probes with designation used is given in the Supplementary Materials.

The task of expanding the number of species detected by the biochip analysis was achieved by the addition of novel probes to the same region of the *gyrB* gene, which corresponds to the species identified in our previous study. Furthermore, probes for *M. leprae* cluster species were added to allow possible use of the test in areas with a noticeable incidence of leprosy. Thus, the biochip consisted of 144 oligonucleotide probes (Table S1), immobilized on a plastic plate [33] in an array with dimensions of 3 by 5 mm (Figure 1).

The fluorescently labeled amplicon of the *gyrB* gene is obtained by single-step asymmetric amplification with Cy5 incorporation during the elongation using Cy5-dUTP substrate. The extended length of probes allowed hybridization with imperfect matches, thus allowing the identification of expanded species spectra by means of recognition of a hybridization profile. The obtained profile was checked against the reference database of profiles, and coefficients of determination for linear regression of pairwise fluorescence signals were calculated. The R^2^ values above 0.9 were considered significant for reliable detection of the species.

The assay was initially validated using laboratory DNA collection and, further, in the settings of clinical antituberculosis centers. The novel profiles with low correlation to known species were added to the reference database, while species identification was performed by sequencing. Thus, during the initial validation and analysis in clinical centers, the spectra of identified species was continuously expanded. The updated database was distributed immediately between centers to facilitate rapid identification on site. Usually, at least two–three profiles from independent samples or independent amplifications and hybridizations were used in the reference database to diminish the impact of fluorescence signal fluctuations.

Finally, the Myco-biochip test could identify 79 mycobacterial species; 68 were identified during the analysis of clinical samples in Russia in 2022–2024 (described below), and 11 species were identified after the analysis of laboratory collection from different sources and years. These include clinical isolates of *M. bulgaricum* sp. nov., *M. marinum*, and *M.* sp. GF 28 from the recent study of isolates from Bulgaria [45], *M. smegmatis*, *M. flavescens*, and *M. saskatchewanense* from the study of clinical isolates published by our group in 2015 [44]. Other DNA samples were from unpublished samples of species, *M. phlei* and *M. intermedium*, obtained from clinical isolates from patients in Moscow, and *M. terrae* and *M. sherrisii*, obtained from unknown sources. For the unpublished samples, species identification was performed by target sequencing of *gyrB* and *rrs* fragments.

Since the diversity of *gyrB* between strains of the species could be significant, several distinct profiles were identified for *M. avium*, *M. intracellulare*, *M. chelonae*, *M. abscessus*, and other species (Figure 1). Separate profiles strictly corresponded to sequence types of the gene, but do not necessarily represent the subspecies due to possible mosaic evolution as was shown for *M. avium* [46,47].

In total, 130 distinct profiles for 79 *Mycobacteria* and 10 species of other genera of *Mycobacteriales* were obtained. The reliability of the species identification method was confirmed by the pairwise analysis of all profiles (Figure S1). The distribution of interspecies correlation was below the threshold of 0.9 with the mode in the range of 0.00–0.05. The intraspecies correlation coefficients between 0.8 and 0.9 were recorded for several pairs of closely related species, *M.* sp. CSUR_Q5927 and *M.* sp. GF74 (R^2^ = 0.83), *M. gilvum* and *M. mephinesia* (R^2^ = 0.83), and between the three members of *M. gordonae* complex, *M. gordonae* itself, *M.* sp. CTRI_14-8773, and *M. paragordonae* (R^2^ = 0.83–0.89).

### 2.2. The Spectra of Mycobacterium Species Identified in Clinical Isolates

The biochip-based assay was implemented in three TB research centers in Russia, located in Moscow, Saint-Petersburg, and Novosibirsk, which are the largest cities in the country. Centers in Novosibirsk and Saint Petersburg collect isolates from regional centers, from their home regions, ‘Novosibirsk oblast’ and ‘Leningrad oblast’, respectively, and also from neighboring territories Karelia, Murmansk, Tomsk, and others. The samples analyzed in Saint-Petersburg represent the whole north–west part of Russia. The Moscow TB center is responsible for Moscow city only; however, its population exceeds 15 million, which is about 10% of the whole population of the country.

In total, 3123 samples from 2211 patients were evaluated. The spectra of nontuberculous mycobacteria found in clinical settings is discussed further using the cases numbers. Multiple isolation from one patient was accounted for in the evaluation of clinical significance of different species and testing the biochip methodology. Both frequencies are provided in Table S2.

The identification of *Mycobacteria* species was successful in 1988 of 2211 cases. The failed identification includes 17 cases with mixed hybridization profiles. Furthermore, in 50 cases, the biochip result was negative, and the target sequencing also failed to obtain a reliable chromatogram. In addition, species identification failed in ten cases, while specific hybridization profiles were obtained. In most of these cases, contamination commonly met in water and molecular biology kits *Microbacterium oxygens* was observed by sequencing of the 16S rRNA gene fragment [48].

Unexpectedly, other species of the order *Mycobacteriales* that do not belong to *Mycobacteria* were found in a substantial number of cases (*n* = 45). They were identified by ‘weak’ hybridization with a small number of significant signals, since no specific probes for species from other orders were planned in the biochip design. However, these profiles were different from *Mycobacteria* profiles (Figure S1). We did not perform the systematic study of these isolates; only a part of them was analyzed by sequencing the 16S rRNA or *gyrB* gene fragments (*n* = 16). Five samples belong to *Nocardia*, 4 *Gordonia*, 3 *Corynebacterium*, 3 *Tsukamurella*, and one sample belongs to genus *Rhodococcus*. In two cases, independent isolates of *Tsukamurella tyrosinosolvens* were found in sequential isolates from the same patients, which confirmed the clinical relevance of the identification of other members of *Mycobacteriales*.

Another category of cases that do not allow one to confirm the infection with nontuberculous mycobacteria is different species isolated from sequential samples of the same patient. One hundred and one of these cases were identified, reflecting the possibilities of mixed infection, colonization of the respiratory tract, or reinfection of a susceptible host [49]. The isolation frequencies in such heterogenic cases repeat the overall prevalence of the species, with *M. avium* (*n* = 54), *M. intracellulare* (*n* = 24), *M. abscessus* (*n* = 20), *M. lentiflavum* (*n* = 12), and *M. kansasii* (*n* = 10) being the most frequent. However, the frequency of identification of other *Mycobacteriales* in these cases was significantly higher (*n* = 10, *p* < 0.0001).

**Figure 2:**
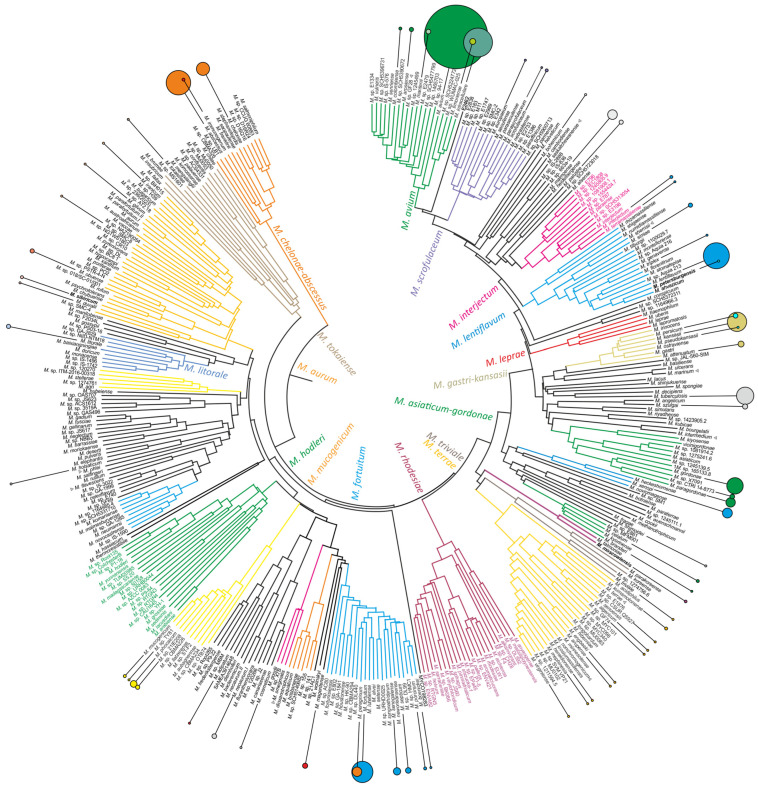
Frequency of *Mycobacteria* species in patients with TB-like disease in Russia. The phylogenetic tree was constructed on the basis of pairwise amino acid identity (AAI) calculation. The squares of callout bubbles are proportional to the number of clinical cases. The novel mycobacterial species identified in the study are given in bold. An additional 11 species that could be identified by Myco-biochip are marked with triangles.

Mycobacterial species were identified in 1988 clinical cases, and as expected, the *M. avium* complex species, *M. avium* (*n* = 927) and *M. intracellulare* (*n* = 189), were the most prevalent nontuberculous mycobacteria identified in 1988 cases, followed by *M. lentiflavum* (*n* = 153), *M. abscessus* (*n* = 136), *M. fortuitum* (*n* = 99), and *M. kansasii* (*n* = 84) (Figure 2). The repeated isolation of the same species in a patient was approximately 26% for *M. avium* (*n* = 251), *M. abscessus* (*n* = 36), and *M. intracellulare* (*n* = 50). Multiple isolations of *M. kansassii* were observed in 51% of the cases (*n* = 43). The counts of *M. lentiflavum* (*n* = 16) and *M. fortuitum* (*n* = 13) were approximately 10%. However, this is an estimate of the lower border of confirmed cases, since other diagnostic criteria, such as symptoms and radiographic evidence, were used [50].

The identified species could be assigned to 25 clusters or complexes (Table 1), in addition to the *M. mageritense*, which is close to the *M. fortuitum* complex, but is rooted deeper on the phylogenetic tree, and has no closely related genomes identified (Figure 1). The first eight most frequent clusters, consisting of 29 species, are responsible for 95% of the NTM identifications.

In 68 patients, *M. tuberculosis* was identified, adding to the total number of tuberculosis cases that were missed by the immunoassay performed after the cultivation step. The developed biochip allows for the differentiation of *M. tuberculosis* sensu stricto from the var. *bovis* by a polymorphism N389N (aac>aat) in the *gyrB* gene. The latter cases identified in TB clinical centers are usually cases of disseminated infection caused by vaccination with *M. bovis* BCG, either neonatal or as a treatment for bladder cancer [51]. We identified two such cases; however, the assay result cannot be used for diagnosis due to the inability to differentiate strains within *M. bovis*, and more specific tests should be used.

Additionally, isolates for five unnamed mycobacterial species were identified (Table 2). In two cases in Saint Petersburg and Novosibirsk, isolates were found to belong to the same species as isolate SMC-8 obtained from the sputum of the patient with lung disease. In five independent cases, we found isolates with *gyrB* sequences identical to that of *M.* sp. 21IE208, isolated from the sputum of a patient in Ireland. This isolate was incorrectly annotated in NCBI as *M. mucogenicum*, since an ANI of 91.3% between strain 21IE208 and *M. mucogenicum* DSM 44625 (SAMN31811490) is below the species delineation threshold. We observed repeated isolation of this species in three of five clinical cases. In one case, after two sequential isolations of *M.* sp. 21IE208, *M. abscessus* was found in the third.

Similarly, five cases of *M.* sp. CTRI_14-8773 identified in Moscow, Saint Petersburg, Karelia, and Murmansk were complemented by another one with sequential isolation of *M. lentiflavum*. *M.* sp. CTRI_14-8773 was incorrectly annotated as *M. gordonae* in NCBI (ANI = 87.3%); however, it represents the distinct species inside the *M. asiaticum-gordonae* cluster, which was closer to *M. paragordonae* (ANI = 89.8%).

Two species were identified only in one of two subsequent isolates obtained from the same patient, *M. engbaeki* and *M.* sp. GF74, followed by the isolation of *M. avium* and *M. peregrinum*. We recently also found these species in clinical samples from Bulgaria [45]; the clinical significance of this finding remains to be explored further. Two more species of doubtful clinical significance, *M. adipatum* and *M. terramassiliense*, were identified in a single sample each; previously, they were isolated from the environment [53,54].

### 2.3. Regional Variation of Mycobacterial Species

**Figure 3:**
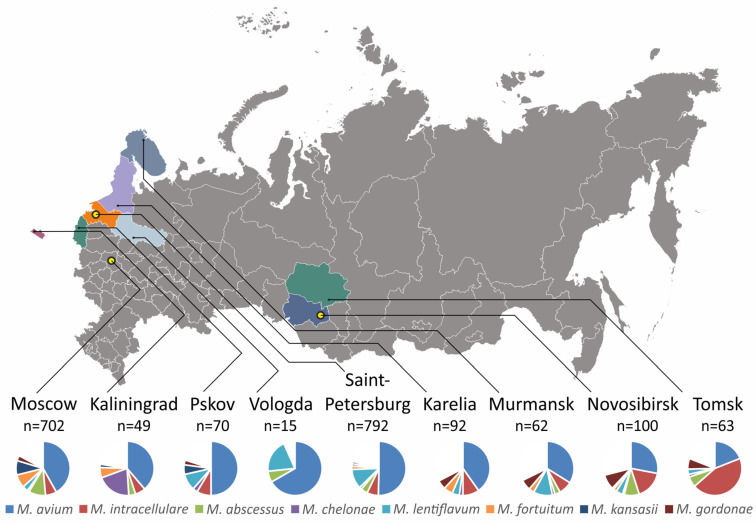
Regional differences in the spectra of NTM isolation in the Russian Federation. Only regions with a significant number of cases were analyzed. The three TB centers responsible for the collection and processing of clinical samples are shown with yellow circles.

We also analyzed regional differences in the spectra of mycobacterial species found in clinical cases. Part of the patient records lacked the place of living, which is mainly caused by the significant level of migration and medical regulation which guides the appointment of foreign citizens. For nine regions, the number of patients was large enough to draw conclusions on the variability of mycobacterial species (Figure 3).

The largest sampling was available for Moscow and Saint-Petersburg with 702 and 792 cases, and statistically significant differences were recorded. The isolation frequencies of *M. abscessus* (71 vs. 32 patients), *M. fortuitum* (56 vs. 16 patients), and *M. kansasii* (60 vs. 11 patients) were higher in Moscow (*p* < 0.0001), while *M. lentiflavum* was found more frequently in Saint-Petersburg (93 vs. 23 patients, *p* < 0.0001).

The number of *M. intracellulare* cases in the Tomsk region was much more frequent than the average (*p* < 0.0001) and exceeded the number of *M. avium* cases in this region, which is a reverse ratio compared to the other regions. It could be proposed that the prevalence of *M. intracellulare* in the neighboring Novosibirsk region is also higher; however, the statistical significance of this finding is lower (*p* = 0.0042).

In Kaliningrad, similar inversion in frequencies was observed for *M. abscessus* and *M. chelonae*, with the latter being more prevalent than the former (10 vs. 2 cases, *p* = 0.0444) and significantly above the average isolation frequency (*p* < 0.0001).

Lastly, *M. gordonae* was found more frequently in Novosibirsk, above the average prevalence (9/86 vs. 61/2107 cases, *p* = 0.0006). All other frequency differences were statistically insignificant.

### 2.4. Whole-Genome Sequencing of Novel Mycobacterial Species

Three isolates, which sequences of *rrs* and *gyrB* fragments had low identity with known species were sequences by the short-reads whole-genome sequences and assembled to contigs level with Shovill. The sequences obtained were deposited in NCBI and used for the Protologger taxonomy pipeline [55], recommended for defining the novel taxonomic units (Table 3 and Table S3).

The genome analysis of strain pU-009, isolated in Moscow, identified *M. talmoniae* as the closest species. Although the *rrs* gene was almost identical for these genomes, the ANI value was only 92%, which is below the 95% threshold for species delineation [56]. *M. talmoniae* is an orphan species that, together with *M. terrae* and *M. triviale* complexes, belongs to the most ancient branches of slow-growing mycobacteria. *M. talmoniae* has been detected in the sputum of patients in Switzerland [57] and the United States [58]. Both belong to the same species observed by genome–genome comparison; the strain from Switzerland was named *M. eburneum* and the latter named *M. talmoniae*. Both names were published according to accepted taxonomic rules in 2017; however, the name *M. talmoniae* has priority [59]. The novel isolate is a second species in a hypothetical significantly distinct *M. talmoniae* complex (Figure 2).

Similarly, the genome of strain pV-006, isolated from a patient in Novosibirsk, is expanding the diversity of the *M. duvalii* cluster of fast-growing mycobacteria consisting of only two species. The *M. duvalii* species itself was isolated from a patient with leprosy in 1912 and was considered the etiologic agent of the disease [60]. The second species, which does not have its own name, was isolated from the sputum of a patient in Korea in 2019, and has 84% homology to *M. duvalii* (accession SAMN20167676). The genome of our discovered isolate is 84% similar to that of *M. duvalii* and 83% similar to the second strain. The genomic assembly of pV-006 isolate had a lower genome size of 5.1 Mbases and number of genes, 4963, in comparison with *M. duvalii*. The latter had a genome size of 5.6 Mbases and 5417 total genes (Acc. No. GCF_002553585.1). A year later, in February 2023, another isolate with an identical hybridization pattern and sequences of *gyrB* and *rrs* fragments was identified at the Saint Petersburg Research Institute of Phthisiopulmonology.

The third isolate, pR-1184, which is supposed to represent a novel species, was obtained from a single sputum sample from the patient in Saint-Petersburg. Sanger sequencing of the *gyrB* fragment revealed 24 mismatches per 411 total length with *M. ahvazicum* (SAMEA103958175), which was supposed to exceed the threshold for species delineation. The same pattern and sequence were also identified in DNA collection from our older study, in the set of isolates from the same Saint Petersburg Research Institute of Phthisiopulmonology [44]. It was misidentified as *M. lentiflavum* based on the sequence of the *rrs* fragment, and the sequence of the *gyrB* fragment of the isolate was deposited in the NCBI nr/nt database under the accession KJ725325.1 [44]. The whole-genome sequencing of the isolate represents the novel mycobacterial species with the closest relative *M. ahvazicum* (ANI = 93.9%) and *M. lentiflavum* (ANI = 91.6%). The pathogenic species *M. ahvazicum* was isolated from respiratory tract and soft tissue biopsies in four patients in 2009 in Iran [61]. The ANI value between *M. ahvazicum* and *M. lentiflavum* is 91.7%, thus these three species comprise the compact cluster inside the *M. lentiflavum* clade (Figure 2).

We propose to name the discovered species *M. moscowiensis*, *M. sibericum*, and *M. petersburgensis*, to indicate the first places of isolation (Table 3).

## 3. Discussion

The developed biochip approach that utilizes the imperfect binding of a genome fragment with oligonucleotide probes provides the continuous expansion of the list of identifiable species by means of a profile recognition algorithm. Samples with a novel hybridization pattern are analyzed by sequencing, and the pattern is added to the database for comparison with the correct annotation. Analysis of more than 3000 samples demonstrated the low cross-correlation of profiles corresponding to different species. A total of 130 different profiles were found at the development and implementation stages, showing the ability to identify 79 distinct mycobacterial species.

The further improvement of the biochip assay described in 2015 [44] was pushed forward by the growing number of mycobacterial species found in clinical samples. We observed nearly identical hybridization profiles for distinct species, identified by sequencing *M. cosmeticum*, *M. neoaurum*, *M. adipatum*, and *M.* sp. SMC-8. The other group of species with an identical profile included *M. terrae*, *M. arosiense*, *M. monacense*, and *M. duvalii* species. It was not unexpected, due to the overall homology of mycobacterial genomes, and the absence of specific probes. However, the used *gyrB* fragment is a robust species-specific marker.

On the contrary, the methods based on the analysis of rRNA genes have the intrinsic limitation based on the horizontal transfer of ribosomal operon and lower evolution rate: not 16S, nor 23S rRNA genes allows the delineation for all the species in the genus [36]. This is correct for any type of detection of specific sequences inside these loci, either hybridization [25,62], real time [28], or sequencing [63,64]. Two commercial assays, widely used throughout the world—GenoType CM and AS—allow the identification of 30 species with the reported limitation of undesired cross-reaction for some rare species [25]. Other more robust genes like *rpoB* [31,32], *gyrB* [41,42,43], ITS fragment [37,38], *hsp65* [39,40], and *secA1* [29] are also used in a large number of developed approaches. However, the number of species identified in a significant number of studies did not exceeded several tens [41,65,66]. Therefore, the large-scale studies that report the extended spectra of species utilize multilocus sequencing [67,68], which is unfeasible for clinical laboratories. Using the developed assay in three antituberculosis clinical centers in Russia during 2022–2024, as high as 68 species were specifically identified, including the three novel ones.

We identified a close phylogenetic neighbor of orphan *M. talmoniae*, located between *M. terrae* and *M. triviale* complexes and the branch including all other slow-growing *Mycobacteria*. The novel species with the proposed name *M. moscowiensis* adds to the argument by Turenne et al. [69] against the proposed splitting of *Mycobacteria* into five genera [70]: the existence of proposed *M. talmoniae* clade between *Mycolicibacter*, *Mycolicibacillus*, and *Mycobacteria [emended]* demands the introduction of the sixth genus. Two other new species, *M. sibiricum* and *M. petersburgensis*, also expand the diversity of *Mycobacteria*, particularly of the *M. duvalii* and *M. lentiflavum* clades. The new mycobacterial species had specific hybridization profiles, allowing its further identification using the developed biochip assay.

The diversity of species identified in TB-suspected cases is not limited to the *Mycobacteria* genus. We also observed species from genera *Nocardia*, *Gordonia*, *Corynebacterium*, *Tsukamurella*, and *Rhodococcus* from the order *Mycobacteriales*. They also have a wax cell wall and are capable of surviving the decontamination procedure. The finding of other acid-fast bacilli in TB-suspected cases is not a new observation; in a recent study by Sun et al. [67], *Burkholderia* and *Actinomyces* were identified in addition to *Nocardia*, *Gordonia*, *Corynebacterium*, and *Tsukamurella*, identified in our study. Although we did not perform systematic analysis of these isolates, the repeated isolation of *Tsukamurella tyrosinosolvens* in two independent cases confirms its clinical relevance. This observation demands further improvement of the molecular assays for species identification in suspected cases of NTM-disease, which is necessary for the diagnosis and prognosis of treatment. Importantly, the differentiation of nontuberculous mycobacteria from *M. tuberculosis* is also a necessary property for an assay, as we found 68 additional cases of tuberculosis (of a total of 2221 cases analyzed) missed by the MPT64 method [71].

Repeated isolation of the same species from the patient’s sputum in sequential samples is an essential diagnostic criterion [50]. Further empowerment of this trait is confirmation of the identity of the species genotype, since ‘genotype change’ was recorded for *M. avium* isolates [72,73], possibly reflecting the reinfection of the susceptible host [49]. However, only a single isolation of the presumable pathogen from bronchial wash or biopsy is sufficient for causality determination [50], which is rather questionable based on the possibility of bacterial or DNA contamination. According to previous reports, we observed cases of sequential isolation of different species [74,75]. The clinical interpretation of these cases is challenging and was beyond the aims of the present study. However, for a significant part of cases, we identified one species in separate samples from one patient, and thus we could propose the clinical significance of the species. In particular, we observed such cases for the unnamed isolates SMC-8, 21IE208, and CTRI_14-8773.

The number of species that cause NTM disease is growing, and not limited to *Mycobacteria* itself. This circumstance demands the rapid dissemination of knowledge about the pathogenicity and resistance of particular species. The molecular methods, based on whole-genome data, provide the most sensitive and rapidly upgradable approach for identification of the pathogen.

## 4. Materials and Methods

### 4.1. Biochip Design and Fabrication

The biochip assay was developed based on a previously described approach [44]. Briefly, the fragment of the *gyrB* gene is amplified and hybridized with the matrix of immobilized oligonucleotide probes, which refer to different mycobacterial species. Multiple sequence alignment of the known 381 species (Zimenkov et al., submitted) was used for the selection of conservative and variable regions for the selection of primer binding sites and for species-specific probes. Three separate probes were selected for each species. In addition to previously described species, probes for *M. abscessus*, *M. phlei*, *M. flavescens*, *M. duvalii*, *M. obiense*, *M. iranicum*, *M. neoaurum*, *M. mageritense*, *M. houstonense*, *M. setense*, *M. fortuitum*, *M. persicum*, *M. conceptionense*, *M. septicum*, *M. mucogemicum*, *M. senuense*, *M. leprae*, *M. lepraemurium*, *M. celatum*, *M. lentiflavum*, *M. simiae*, *M. saskatchewanense*, *M. interjectum*, *M. colombiense*, and *M. mantenii* were also selected. The final layout of the biochip is presented in Figure 1.

Biochips were manufactured as previously described (38). The biochip was made up of 144 meaningful elements with immobilized oligonucleotides, three marker cells (M) for accurate positioning (image acquisition) with processing software, and four empty gel elements (0) needed to calculate the reference fluorescence intensity (background). The biochip hybridization and analysis of fluorescence was carried out using the ImaGeWare v.3.5. software (Biochip-IMB LLC, Moscow, Russia) as previously described (38).

The profile obtained was checked against the reference database of profiles and coef-ficients of determination for linear regression of pairwise fluorescence signals were calculated [76]. The R^2^ value above 0.9 was considered significant for the reliable detection of the species. The value was derived from the analysis of the hybridization profiles obtained during the study and is relevant for all anti-tuberculosis centers with implemented biochip technology.

### 4.2. Processing of Clinical Isolates

Patients suspected of tuberculosis were routinely examined in clinical centers. Samples (sputum, bronchoalveolar lavage (BAL), gastric washes (in children), and biopsy materials) were collected, decontaminated, and divided for microscopy, cultivation, and molecular assays. A smear microscopy for acid-fast bacilli (Ziehl-Neelsen or fluorescence) was performed from the specimens. The strains were isolated using solid (Loewenstein-Jensen) and liquid media using the automated Bactec MGIT 960 system (Becton Dickinson Microbiology Systems, Cockeysville, MD, USA) according to the relevant standard operating procedures of the national and international guidelines [77,78]. Acid-fast samples with a negative for *M. tuberculosis* cultures by immunochromatographic Bioline™ TB Ag MPT64 (Abbott Point of Care Inc., Abbot Park, IL, USA) were supposed to contain nontuberculous mycobacteria and further studied by microbiological and biochip analysis. DNA samples with unknown specific hybridization profiles were analyzed by Sanger sequencing as described previously [45].

### 4.3. Phylogenetic Analysis and Target Sequencing

The phylogenetic tree for order *Mycobacteria* was obtained based on the average amino acid identity distance metrics. Details of the phylogenetic analysis are to be published separately (Zimenkov et al., submitted). Briefly, all assembled genomes of the genus were downloaded from NCBI and the pairwise genome–genome distance matrix were calculated using the ezAAI algorithm [79]. Initial species and subspecies discrimination were performed using the 95% and 98% thresholds of the ANI value, calculated using the fastANI approach [80]. Four genomes of *Nocardia*, *Corynebacteria* were used as an outgroup. The 381 core genomes representing different species were used for identification by target sequencing. The identification of species was based on the local implementation of a BLAST search using downloaded genomes.

The value of 20 mismatches per 300 bp of the *gyrB* fragment was estimated to correspond to 95% of the species delineation threshold. Thus, isolates with a number of mismatches above were considered to represent a novel specie, and further whole-genome sequencing was performed. Thus, three novel species were identified, sequenced and added to the phylogenetic analysis. Distances were imported into MEGA 11 software [81] and the nearest-neighbor phylogenetic tree was constructed (Figure 2).

### 4.4. Whole-Genome Sequencing and Bioinformatic Analysis

MGIEasy Universal DNALibrary Prep Set (MGI Tech, Shenzhen, China) according to the manufacturer’s protocol. DNA fragmentation was performed by ultrasonication using Covaris S-220 (Covaris, Inc., Woburn, MA, USA) with an average fragment length of 250 bp. The concentrations of DNA libraries were measured using Qubit Flex (Life Technologies, Carlsbad, CA, USA) with the dsDNA HS Assay Kit (Invitrogen, Waltham, MA, USA) following the manufacturer’s protocol. The quality of the prepared libraries was evaluated using Bioanalyzer 2100 with the High Sensitivity DNA Kit (Agilent Technologies, Santa Clara, CA, USA) according to the manufacturer’s instructions. The enriched library pools were further circularized and sequenced with paired-end approach using DNBSEQ-G400 with the DNBSEQ-G400RS High-throughput Sequencing Set PE100 following the manufacturer’s instructions (MGI Tech, Shenzhen, China), with an average coverage of 500. Fastq files were generated using basecall Lite software (ver. 1.0.7.84) from the manufacturer (MGI Tech, Shenzhen, China).

Genomes were further assembled to contigs from Fastq files using Shovill (https://github.com/tseemann/shovill, accessed on 30 July 2024) at the Galaxy web platform public server (https://usegalaxy.org, accessed on 30 July 2024) [82]. Genome data were deposited in the NCBI SRA and Genome databases (PRJNA1138261).

Genome–genome distances were estimated using the average nucleotide identity (ANI) value using the FastANI approach [80]. Genomes of presumably novel species were analyzed using the Protologger [55] through the online portal (https://protologger.bi.denbi.de/, accessed on 1 August 2024). The formal description of the new species was in compliance with the requirement of the International Code of Nomenclature of Prokaryotes (2022 Revision) [83].

## Figures and Tables

**Table 1:** Distribution of identified species joined in phylogenetic clusters of *Mycobacteria*.

Cluster or Complex	Growth Rate	Species in Cluster	Total Cases	Cases with Repeated Isolation
*M. avium*	slow	6	1135	306
*M. chelonae-abscessus*	rapid	3	177	38
*M. lentiflavum*	slow	4	159	17
*M. fortuitum*	rapid	6	141	15
*M. gastri-kansasii*	slow	4	98	45
*M. asiaticum-gordonae*	slow	3	86	6
*M. tuberculosis*	slow	1	68	-
*M. malmoense*	slow	2	25	6
*M. xenopi*	slow	1	22	5
*M. mucogenicum*	rapid	4	17	4
*M. terrae*	slow	9	8	0
*M. neoaurum*	rapid	3	7	0
*M. szulgai*	slow	1	6	3
*M. aurum*	rapid	3	5	3
*M. chubuense*	rapid	1	4	1
*M. shimodei*	slow	3	4	1
*M. scrofulaceum*	slow	3	4	2
*M. litorale*	rapid	1	4	1
*M. duvalii*	rapid	2	3	0
*M. interjectum*	slow	2	3	0
*M. talmoniae*	slow	1	2	0
*M. triviale*	slow	1	1	0
*M. bohemicum*	slow	1	1	0
*M. tokaiense*	rapid	1	1	0
*M. elephantis*	rapid	1	1	1
(*M. mageritense*)	rapid	1	6	1

**Table 2:** Identified isolates that belong to unnamed species of *Mycobacteria*.

Cluster	Isolate	Sample	Reference	Total Cases	Cases with Repeated Isolation
*M. aurum*	SMC-8	SAMN20167551	-	2	1
*M. mucogenicum*	21IE208	SAMN31811490	-	4	3
*M. mucogenicum*	TY81	SAMD00239442	-	1	
*M. terrae*	CSUR_Q5927 *	SAMN32979486	-	1	
*M. asiaticum*	CTRI_14-8773	SAMN04123344	[52]	5	1

*—proposed name *M. polyniensis.*

**Table 3:** Whole-genome sequence analysis of the novel *Mycobacterium* species.

Strain Name	pU-009	pV-006	pR-1184
Proposed name	*M. moscowiensis*	*M. sibiricum*	*M. petersburgensis*
Genome accession	GCA_041287095.1	GCA_041287115.1	GCA_041287235.1
Assembly (bp)	5,730,899	5,120,508	5,944,740
GC (%)	68.44	68.01	66.1
Genes	5538	4963	5632
CDSs	5486	4906	5578
Pseudo Genes	109	94	83
RNA	52	57	54
Closest species	*M. talmoniae*	*M. duvalii*	*M. lentiflavum*
ANI (%)	92.11	84.20	91.60

## Data Availability

Genome data were deposited to NCBI SRA and genome databases (PRJNA1138261), sample accession are available from the corresponding table in Supplementary Materials. Oligonucleotide sequences used for biochip fabrication are given in the Supplementary Table.

## Supplementary Materials

The following supporting information can be downloaded at: https://www.mdpi.com/article/10.3390/ijms252313200/s1.
