# Supplementary material for: Biochip-Based Identification of Mycobacterial Species in Russia
Source: Int J Mol Sci. 2024 Dec 8;25(23):13200. doi: 10.3390/ijms252313200 (PMC11642025; doi:10.3390/ijms252313200)
Supplement: Supplementary file 1 [file ijms-25-13200-s001.zip › ijms-3330839-supplementary.pdf]

Figure S1.

Pairwise correlation analysis of fluorescence profiles of mycobacterial species. (A) Coefficient of determination matrix of 120 hybridization profiles above the value of 0.6. Species and sequence types are ordered according the phylogenetics of *Mycobacteria*. Rectangles with thin borders are pointing to correlation of sevral profiles for the same specie, thick border rectangles are corresponding to the main complexes. (B) The distribution of interspecie coefficients of determination.

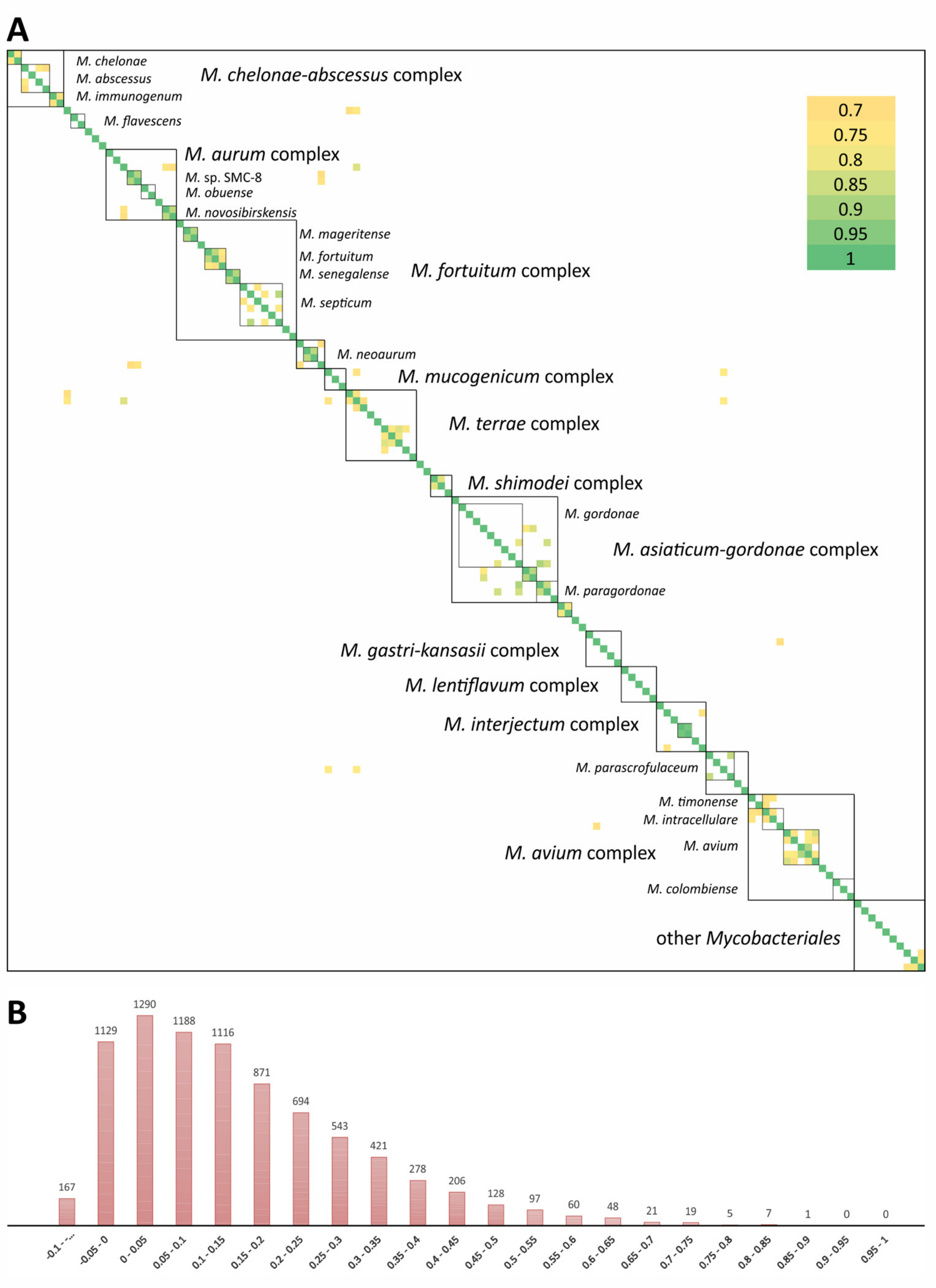

Table S1.

Oligonucleotide probes used for biochip production.

No	ID	Sequence
1	mtb1	GCAAGCCCGTATCGCG
2	mtb2	CGACATCGGTGGATTGCCC
3	mtb3	CGTTCCACGGATCCGCG
4	bov1	TGTAACGAACAGCTGACC
5	bov2	TGTAATGAACAGCTGACC
6	can	CGAAAGTTGTTGTGAACA
7	avi1	CGGTTTCATCAGCGCAGGC
8	avi2	AGAGCGCCACCGACCTGGG
9	avi3	GTCGACCGACCCGCGC
10	par1	CGGTTTCGTCAGCGCAGG
11	par2	AGAGCGCAACCGACCTGG
12	par3	TCGACCGATCCGCGCAA
13	104-1	CGCATTGCCGCCCGC
14	104-2	AGAGCGCAACTGACCTGGG
15	104-3	CCGCTCGACCGACCCG
16	404-1	CGTCAGCACAGGCCCGC
17	404-2	GACCTGGGCGGGC
18	404-3	CTGGGCGGGCTGC
19	int1	CGCAGGCCCGGATCGC
20	int2	AGCGCCACCGATCTGGG
21	int3	CGACGGATCCGCGCAAGTC
22	scr1	GCTCAGGCGCGCATTGC
23	scr2	GGTCTGCCCGGGAAGCTG
24	scr3	CGCGGAAATCGGAACTGTATGT
25	asi1	CGTCCGCACAGGCCCG
26	asi2	CGCGACCGATTTGGGC
27	asi3	CGTTCCACCGACCCGCG
28	gas1	GCGCAGGCCAGGATTGC
29	gas2	GGACTACCGGGCAAGTTGGC
30	gas3	TCGACCGACCCCCGTAAGTC
31	gor1	GCCCAGGCGCGGATCG
32	gor2	GACCTCGGCGGCCTGC
33	gor3	GATCCCCGCAAATCCGAACT
34	kan1	TCGGCGCAAGCACGCATT
35	kan2	TGGGCGGACTACCTGGCAA
36	kan3	CTGCCGCTCGACCGACC
37	mal1	GCCCAGGCCCGAATCGC
38	mal2	CCGGGTAAGCTCGCAGACT

39	mal3	CACCGACCCGCGAAAGTC
40	mar1	CGCAGGCACGTCTCGC
41	mar2	CGATCTCGGTGGGCTGCC
42	mar3	CGCTCGACAGATCCGCGT
43	sim1	CCCAGGCCCGCATTGC
44	sim2	GAGTGCTACGGATTTGGGTGG
45	sim3	TCCGCGGAAGTCTGAGCTGT
46	szu1	GGCACAGGCGCGTATCG
47	szu2	AAGAGCGCTACCGATCTCG
48	szu3	GGTGGAAGGGGACTCGGC
49	xen1	GCCGCGCGAAAAGCCC
50	xen2	AGACTGCAACAGACATCGGAGGA
51	xen3	CGCGAAAATCCGAACTGTA
52	che1	GGTTTCGTCGGCACAGGC
53	che2	AGCTGGTCCGCCGCAAG
54	che3	TCCACCGATCCGTCGAAGTC
55	for1	CAGGCGCGCGCGGC
56	for2	GGCGCGTGATCTGGTGC
57	for3	TCCACCGATCCGAGCAAGTC
58	sme1	CGGATCGCGGCGCGTA
59	sme2	CGACATCGGTGGGTTGCC
60	sme3	GAGCAAGTCGGAGCTGTATGTGG
61	abs-mal1	AGACCAAGCTTGGCAACACC
62	abs-mal2	GGAACTGTATGTAGTGGAGGGC
63	abs-mal3	GACTCGGCTGGCGGCT
64	abs-bol1	ACCGAGGTGAAGTCGTTTGTGC
65	abs-bol2	TATCGGCGGACTGCCGG
66	abs-bol3	GAGCGGTCGTGACTCGATGT
67	abs1	AGACCAAGCTCGGCAACACC
68	abs2	ATATCGGCGGGCTGCC
69	abs3	GCCGTTCGACAGACCCGTC
70	phl1	GCCAACCCAGCTGAGGCT
71	phl2	CAAGTGCGAGCTGTACGTGG
72	phl3	GGGCGGTTCAGCCAAGAG
73	fla1	CGAGCAGATAGGCCACTGGC
74	fla2	TAAGGCCCGGGATCTGGTG
75	fla3	CGATTGTCGGTCTACGGATCCG
76	duv1	CTTCGTGCAGCGGATCTGCAA
77	duv2	CCAAAACGGTGATCAACAAGGC
78	duv3	GACTGCCGGTCCACCGAC
79	obu1	GAACAGCTCACACACTGGTTCGAG
80	obu2	GGTCTCGTCCGCGCAGG
81	obu3	CGGATCCCACCAAGTCGGAA

82	ira1	GCAACGAGCAGATGACACACTGG
83	ira2	GTATTGCGGCTCGTAAGGCG
84	ira3	TGCGGCGGAAGAGTGCGA
85	nea1	GAACAAGGCAGTCTCGTCGGC
86	nea2	GTGAGCTGGTCCGGCGTAAG
87	nea3	GGTCTGCCAGGCAAGCTC
88	mag1	AGCAGCTGACACACTGGTTCG
89	mag2	TTCCTCCGCTCAGGCGC
90	mag3	GGATCCGCGTAAGTCCGAAC
91	mag2.1	GCGGCCGTTCGCTCGTTT
92	mag2.2	CAACCCGGTCGAAGCCAA
93	mag2.3	GCGCGTATTGCGGCGC
94	hou1	CAACGAGCAGATCAGCCACTG
95	hou2	GAGGCGGCTCGTAAGGCG
96	hou3	GCCACCGAGATCGGCGG
97	set1	AGGCGGTTTCATCAGCACAGG
98	set2	GTGAACTGGTCCGGCGTAAGA
99	set3	GCCTGCCAGGCAAGCTG
100	for4	AGCCATTGGCTCGAGGCC
101	for5	GCGCGTGATCTGGTGCG
102	for6	TCCGAGCTGTACGTGGTGGA
103	per1	TCAACAAAGCAGTTTCATCGGCA
104	per2	TGAGCTGGTCCGGCGCAA
105	per3	TGCCGCTCTACCGATCCGA
106	con1	AAGTCGTTCGTTCAGAAGATCTGC
107	con2	GGCCAATCCAGCTGAAGCG
108	con3	TCGGCGGTTTGCCCG
109	sep1	GAACTGGTCCGGCGCAAG
110	sep2	ATCGGCGGACTGCCGG
111	sep3	TGCCGCTCTACCGATCCG
112	muc1	GCCAACCCAGCCGAGG
113	muc2	AGGCCCGTGTCGCCG
114	muc3	CGACCCGACGAAGTCGGAA
115	sen1	CCACTGGCTCGAGGCC
116	sen2	AAGCTGGCAGACTGCCGT
117	sen3	GGTGGAAGGGGATTCGGCC
118	lep1	AATGAGCAACTTATTCACTGGTTTGAA
119	lep2	AGATGCGAAAGCGGTTGTGAATAAG
120	lep3	TGGACTTCCTGGAAAACTTGCC
121	ler1	GCAATGAGCAATTAATCCACTGGTTTGAA
122	ler2	ACTTCCTGGCAAACTTGCTGATTG
123	ler3	TGAACTGTATGTAGTCGAAGGTGATTCG
124	cel1	TCGGCAACACTGAAGTCAAGTCC

125	cel2	CCAATCCCAGCGACGCC
126	cel3	CAACGGATATAGGCGGGCTG
127	len1	CGTGCAGCGGGTCTGC
128	len2	GCCCGGTAAACTGGCCGATT
129	len3	CCACGGATCCGAAGAAATCGGAACT
130	sim1	CGAACAACTCACTCACTGGTTCGAG
131	sim2	GTAAGAGTGCTACGGATTTGGGTG
132	sim3	GGGTGGGTCGGCGAA
133	sas1	GAGGTCAAGTCATTCGTCCAGAAG
134	sas2	ACCGACCCTCGTAAGTCGG
135	sas3	TGGTGGAAGGAGATTCGGCC
136	inj1	GAGGTCAAGTCCTTCGTGCAGAAG
137	inj2	GTAAGGCCCGCGAGCTG
138	inj3	TATGTGGTCGAGGGAGATTCGGC
139	col1	CTGCAACGAACAACTCACCCACT
140	col2	ACGCCAAGGTCGTGGTGAAC
141	col3	GAGTGCCACCGATTTGGGC
142	man1	GAAGTCAAGTCATTCGTGCAGAAG
143	man2	CGCGCGAAAGGCGC
144	man3	GACTGCCGATCCACCGAC

Table S2.

The list and frequencies of identied species.

Cluster	Specie	Samples	Cases	Cases with repeated isolation	TB centers			Regions								
					Mosow	Saint Petersburg	Novosibirsk	Moscow	Kalinigrad	Pskov	Vologda	Saint Petersburg	Karelia	Murmansk	Novosibirsk	Tomsk
M. chelonae	*M. chelonae*	47	39	2	8	29	2	8	10	2		11	2	1	1	1
M. chelonae	*M. abscessus*	216	136	36	71	44	21	71	2		1	32		2	9	4
M. chelonae	*M. immunogenum*	2	2			2				1		1				
M. tokaiense	*M. tokaiense*	1	1	0		1						1				
M. elephantis	*M. holsaticum*	1	1	1		1			1							
M. litorale	*M. monacense*	6	4	1	1		3	1								
M. aurum	*M. iranicum*	1	1	1		1						1				
M. aurum	*M. gilvum*	2	2	1	1	1		1				1				
M. aurum	*M. sp.* SMC-8	3	2	1		1	1						1			
M. chubuense	*M. obuense*	6	4	1		3	1					1	2			1
M. duvalii	*M. duvalii*	1	1	0	1			1								
M. duvalii	*[M. sibiricum]*	2	2	0		1	1		1						1	
orphan	*M. mageritense*	10	6	1		6				1		2	1			
M. fortuitum	*M. peregrinum*	22	20	0	1	5	14	1	1			1	1	1	2	1
M. fortuitum	*M. fortuitum*	121	99	13	56	30	13	56	3			16	5	2	2	
M. fortuitum	*M. senegalense*	18	12	1	4	6	2	4	1			4		1		1
M. fortuitum	*M. septicum*	7	7	0	5	1	1	5	1							
M. fortuitum	*M. boenickei*	1	1	1		1						1				
M. fortuitum	*M. porcinum*	6	2	0	1		1	1								
M. neoaurum	*M. cosmeticum*	1	1	0	1			1								
M. neoaurum	*M. neoaurum I*	6	5	0	3	1	1	3				1			1	
M. neoaurum	*M. adipatum*	1	1	0		1						1				
M. mucogenicum	*M. sp.* 21IE208	7	4	3		4						3	1			
M. mucogenicum	*M. phocaicum*	13	10	1	4	4	2	4	1			2	1			1
M. mucogenicum	*M. sp.* TY81	1	1	0	1			1								
M. mucogenicum	*M. mucogenicum*	3	2	0		1	1					1				
M. terrae	*M. virginiense*	1	1	0		1										
M. terrae	*M. mephinesia*	1	1	0		1							1			
M. terrae	*M. heraklionense*	1	1	0		1						1				
M. terrae	*M. arupense*	1	1	0	1			1								
M. terrae	*M. engbaeki*	1														
M. terrae	*M. algericum*	2	1	0		1							1			
M. terrae	*M. sp.* GF74	1														
M. terrae	*M. kumamotonense*	2	2	0		2							2			
M. terrae	*M. sp.* CSUR_Q5927	1	1	0		1						1				
M. triviale	*M. triviale*	1	1	0	1			1								
M. talmoniae	*[M. moscowiense]*	2	2	0	1	1		1	1							
M. shimodei	*M. branderi*	1	1	0		1						1				
M. shimodei	*M. celatum*	3	2	0	1	1		1						1		
M. shimodei	*M. shimoidei*	4	1	1	1			1								
M. xenopi	*M. xenopi*	34	22	5	8	11	3	8				8	1	1	1	
M. asiaticum	*M. gordonae*	69	61	2	22	23	16	22		2		11	5	4	9	4
M. asiaticum	*M. sp.* CTRI_14-8773	6	5	0	1	4		1				2	1	1		
M. asiaticum	*M. paragordonae*	24	20	4	2	17	1	2		2		9	3	2	1	
M. szulgai	*M. szulgai*	12	6	3		4	2			1		2			2	
M. tuberculosis	*M. tuberculosis*	80	68	-	15	28	25	15	1	3		8	2	1		1
M. kansasii	*M. attenuatum*	10	8	0		8			1			4	2	1		

M. kansasii	*M. pseudokansasii*	1	1	0		1								1		
M. kansasii	*M. kansasii*	196	84	43	60	16	8	60	1	4		11			1	
M. kansasii	*M. persicum*	9	5	2	3	1	1	3						1	1	
M. lentiflavum	*M. lentiflavum*	182	153	16	23	124	6	23		7	3	93	4	7	4	2
M. lentiflavum	*[M. peterburgense]*	1	1	0		1						1				
M. lentiflavum	*M. triplex*	2	1	0			1									
M. lentiflavum	*M. simiae*	4	4	0	1	3		1				2				
M. interjectum	*M. interjectum*	4	2	0		2						2				
M. interjectum	*M. terramassiliense*	1	1	0		1						1				
M. malmoense	*M. palustre*	5	4	0		4						2	2			
M. malmoense	*M. malmoense*	34	21	6	1	19	1	1		1	1	14	2	1	1	
M. bohemicum	*M. bohemicum*	1	1	0		1						1				
M. scrofulaceum	*M. parascrofulaceum*	4	1	0	1			1								
M. scrofulaceum	*M. scrofulaceum*	4	2	2	1	1		1				1				
M. scrofulaceum	*M. europaeum*	1	1	0	1			1								
M. avium	*M. timonense*	14	8	2	3	2	3	3				2			1	
M. avium	*M. intracellulare*	320	189	50	47	85	57	47	3	6		54	9	5	17	28
M. avium	*M. avium*	1411	927	251	294	559	74	294	19	35	10	399	37	21	28	12
M. avium	*M. mantenii*	6	5	1	1	4		1				3		1		
M. avium	*M. arosiense*	6	4	1		3	1					3				1
M. avium	*M. colombiense*	3	2	1	1	1		1						1		
Mycobacteriales		56	45		5	20	20	5	1	2		10	3	2	2	1
Negative and failed		67	60	0	5	44	11	5	1	3		32		1	1	
Mixed		29	17		8	7	2	8				6		1	1	1
Cases with species switch		-	101	-	36	37	28	36				28	3	2	14	4
**Total**		**3123**	**2211**	**454**	**702**	**1185**	**324**	**702**	**49**	**70**	**15**	**792**	**92**	**62**	**100**	**63**
Mycobacteria		**2971**	**1988**	**454**	**648**	**1077**	**263**	**648**	**47**	**65**	**15**	**716**	**86**	**56**	**82**	**57**

Table S3.

Properties of genomic sequences of the new mycobacterial species.

Strain Name		pR-1184	pV-006	pU-009 (pUA-109)
Proposed name		*M. petersburgensis*	*M. sibiricum*	*M. moscowiensis*
SRA accession		SRR30017327	SRR30017325	SRR30017326
Genome accession		GCA_041287235.1	GCA_041287115.1	GCA_041287095.1
Shovill report				
Total Reads (#)		13,013,820	5,797,930	5,633,487
Assembly (bp)		5,944,740	5,120,508	5,730,899
Estimated genome size (bp)		6,035,093	5,782,606	6,379,160
Genome Coverage		100X	100X	100X
Reads len+		125	125	125
Contigs (#)		69	86	105
Protologger report				
Genome Completeness (%)		100	99.91	100
Contamination (%)		0.15	0.19	0.98
GC (%)		66.1	68.01	68.44
CDS (#)		5564	4885	5448
16S rRNA sequence		not novel	not novel	not novel
	Closest specie	*M. lentiflavum*	*M. duvalii*	*M. talmoniae*
	Genbank accession	AF480583	U94745	KX008970
	Identity (%)	99.86	99.13	99.28
Genomic sequence		novel	novel	novel
	Closest specie	*M. lentiflavum*	*M. duvalii*	*M. talmoniae*
	ANI (%)	91.60	84.20	92.11
NCBI wgs				
Closest specie		*M. ahvazicum*	*M. duvalii*	*M. talmoniae*
Genbank accession		GCF_900176255.2	GCF_002553585.1	GCF_001854525.1
ANI (%)		93.91	84.27	92.95
